# Extraction and Depolymerization of Lignin from Different Agricultural and Forestry Wastes to Obtain Building Blocks in a Circular Economy Framework

**DOI:** 10.3390/polym16141981

**Published:** 2024-07-11

**Authors:** María Ángeles Fontecha-Cámara, Irene Delgado-Blanca, María Mañas-Villar, Francisco José Orriach-Fernández, Belén Soriano-Cuadrado

**Affiliations:** Andaltec, Plastic Technological Center, 23600 Martos, Spain; mangeles.fontecha@andaltec.org (M.Á.F.-C.); irene.delgado@andaltec.org (I.D.-B.); maria.manas@andaltec.org (M.M.-V.); francisco-jose.orriach@andaltec.org (F.J.O.-F.)

**Keywords:** lignin, extraction, depolymerization, microwave, ultrasonic, GNPs

## Abstract

Large amounts of agri-food waste are generated and discarded annually, but they have the potential to become highly profitable sources of value-added compounds. Many of these are lignin-rich residues. Lignin, one of the most abundant biopolymers in nature, offers numerous possibilities as a raw material or renewable resource for the production of chemical products. This study aims to explore the potential revalorization of agricultural by-products through the extraction of lignin and subsequent depolymerization. Different residues were studied; river cane, rice husks, broccoli stems, wheat straw, and olive stone are investigated (all local wastes that are typically incinerated). Traditional soda extraction, enhanced by ultrasound, is applied, comparing two different sonication methods. The extraction yields from different residues were as follows: river cane (28.21%), rice husks (24.27%), broccoli (6.48%), wheat straw (17.66%), and olive stones (24.29%). Once lignin is extracted, depolymerization is performed by three different methods: high-pressure reactor, ultrasound-assisted solvent depolymerization, and microwave solvolysis. As a result, a new microwave depolymerization method has been developed and patented, using for the first time graphene nanoplatelets (GNPs) as new promising carbonaceous catalyst, achieving a 90.89% depolymerization rate of river cane lignin and yielding several building blocks, including guaiacol, vanillin, ferulic acid, or acetovanillone.

## 1. Introduction

The agri-food industry often generates by-products as a result of its activities. These by-products are generally considered low-value waste and are discarded or incinerated on-site for logistical and economic reasons. However, they may contain valuable components or active ingredients relevant to other sectors. This scenario underscores the role of the circular economy, which aims to mitigate greenhouse gas emissions and optimize resource utilization through the recovery and valorization of waste and by-products. In this context, lignin is a highly undervalued agricultural waste product. Lignin is one of the most abundant aromatic biopolymers in nature, providing structural rigidity to plant tissues, and is present in all vascular plants. It forms part of the cell wall alongside cellulose and hemicellulose, organized at the nano-structural level into lignin–carbohydrate networks. The composition and distribution of these components vary depending on the plant type. For example, wood typically contains lignin (15–25%), cellulose (38–50%), and hemicellulose (23–32%). Lignin is increasingly recognized as an affordable and renewable resource with potential industrial applications [1], with an estimated annual production of 5–36 × 10^8^ tonnes.

Lignin is a copolymer primarily derived from three basic phenylpropane monomer units (monolignols): p-coumaryl alcohol, coniferyl alcohol, and sinapyl alcohol. It serves as a valuable biobased feedstock both for direct applications and as a source of chemicals following depolymerization. On the one hand, lignin polymers find direct use in producing lignosulfonates, carbon materials, polymer resins, and adhesives, and as a copolymer or additive in new materials and composites [2,3]. For instance, incorporating lignin into polymers, such as poly(lactic acid) (PLA) [4] or poly(ethylene) (PE) [5], has been proposed to enhance their thermal and mechanical properties, as well as introduce novel functionalities like antioxidant, antimicrobial, and barrier properties [6,7]. On the other hand, lignin’s monolignol structure makes it a potential source of high-value aromatic compounds in the petrochemical industry, which commonly uses fossil resources as feedstock [8]. Due to the gradual depletion of oil reserves, the study and application of sustainable and environmentally friendly alternative sources of energy and chemicals are attracting significant research interest. Biomass presents itself as one of the renewable and affordable solutions to reduce our dependence on oil, highlighting lignin as a promising and valuable source of a wide variety of aromatic compounds and hydrocarbons that can be used in various ways in the chemical industry [9,10]. 

Traditionally associated with the paper industry, lignin extraction yields several commercial lignin types based on the chemical process applied to the lignocellulosic raw materials [11]. (a) Kraft lignin is produced by the kraft or sulfate process, which involves biomass treatment with NaOH and Na_2_S at 150–170 °C, producing as a final by-product a dark solution called “black liquor”. Lignin is recovered from the black liquor by means of acidic precipitation. (b) Lignosulfonates are produced by the sulfite process, based on the dissociation and sulfonation of the lignin by the ion HSO_3_^−^ at 125–150 °C. (c) The soda process is often used to extract lignin from non-wood matrices like straw or bagasse through NaOH digestion and subsequent acidic precipitation of the obtained lignin. (d) Organosolv lignin is obtained by using organic solvents with acid or basic catalysis at temperatures up to 200 °C. In addition to these conventional processes, new promising methods are emerging as alternatives with lower environmental costs. The use of alternative solvents as recyclable ionic liquids shows high yields in lignin extraction [12], while deep eutectic liquids are also a biodegradable and economical option for ionic liquids [13,14]. All of these methods aim to dissolve lignin in order to separate it from the biomass, but other techniques propose to isolate lignin by means of dissolving cellulose and hemicellulose (generally with acids), obtaining lignin as a solid residue. 

Furthermore, lignin depolymerization represents a significant challenge and opportunity in biomass valorization. Lignin depolymerization typically involves breaking α- and β-aryl ether (C-O) bonds. Biological and chemical processes have been described for the degradation of both phenolic and non-phenolic lignin polymer units [15]. Different strategies have been reported for the chemical depolymerization of lignin. (a) Hydrolysis with basic [16] or acid [17] catalysis produces low-molecular-weight compounds, which represent a variety of high value-added chemical products, including a group of phenolic compounds such as vanillin, cresols, catechol, and guaiacol. Due to lignin solubility in alkaline conditions, hydrolysis with alkaline homogeneous catalysis is the main strategy conventionally used to achieve lignin depolymerization. Labidi et al. [18] evaluated the activity of different basic homogeneous catalysts (NaOH, KOH, LiOH, K_2_CO_3_, and Ca(OH)_2_) in lignin hydrolysis; the best yields were obtained with sodium hydroxide (20% by weight of the organic phase). (b) Reductive depolymerization or hydrogenolysis with H_2_ is also used, usually accelerated by acid catalysis [19,20]. This process produces aromatic compounds and highly hydrogenated hydrocarbons. (c) Oxidative depolymerization uses oxidants, such as O_2_ [21] or H_2_O_2_ [21], and a metal-based catalyst to break down lignin into aromatic compounds. 

Most of these strategies for lignin extraction and depolymerization require high temperature and pressure conditions. Alternative approaches use microwave or ultrasound energies, enabling the production of aromatic monomers while reducing the operating conditions [22]. The sonomechanical energy of ultrasound promotes the disintegration of solute particles and enhances solvent accessibility by increasing the surface area of the reagents [21]. Ultrasound also generated hydrodynamic shear forces in the aqueous phase due to the rapid collapse of microbubbles formed during cavitation. As a result, high temperatures and pressures arise inside the collapsing cavitation bubbles (approximately 5000 °C and 2000 atm, respectively), leading to the formation of free radicals and other reactive species. Under these conditions, homolysis or partial cleavage of lignin–carbohydrate bonds can occur, resulting in hemicellulose and lignin separation during lignin extraction [22,23]. Furthermore, depolymerization processes are promoted by the formation of new macroradicals, which can react with each other [24,25,26]. On the other hand, microwave energy causes the rotation of polar molecules and ionic conduction, generating large amounts of heat but avoiding physical contact with the heat source. This allows faster and more economical degradation of lignin compared to traditional methodologies [27]. Microwave depolymerization of lignin has been investigated with and without catalysis [28]. Some studies have related the use of metallic salts as catalysts to the selectivity of the reaction. Zhu et al. described a microwave method using ferric sulfate as a catalyst that selectively cleaves the Cα-Cβ bonds and increases the yield of phenolic monomers [29]. Others works suggest the use of acid catalysis with formic acid [30] or sulfuric acid [31]. Carbonaceous materials are also alternative catalysts, such as activated carbon, charcoal, or graphite [32,33,34].

This study aims to explore the potential revalorization of agricultural by-products through the extraction of lignin and subsequent depolymerization. Specifically, river cane, rice husks, broccoli stems, wheat straw, and olive stone are investigated, five local wastes that are typically incinerated. Traditional soda extraction, enhanced by ultrasound, is applied, comparing two different sonication methods. Once lignin is extracted, depolymerization is performed by three different methods: high-pressure reactor, ultrasound-assisted solvent depolarization, and microwave solvolysis. As a result, a new microwave depolymerization method has been developed and patented, using graphene nanoplatelets (GNPs) as a new promising carbonaceous catalyst.

## 2. Materials and Methods

### 2.1. Materials

Five samples of agricultural by-products or forest wastes, typically incinerated, were received from consortium partners involved in the Agromatter project for valorization. Aitex (Textile Industry Research Association, Alicante, Spain), CTAEX (National Agri-Food Technology Centre, Extremadura, Spain), and CTNC (National Technology Centre for Canning and Food, Murcia, Spain) were the partners. 

These wastes include river cane, rice husks, broccoli, wheat straw, and olive stones. The reagents used in this study, such as sodium hydroxide (NaOH), hydrochloric acid (HCl), sulfuric acid (H_2_SO_4_), graphene nanoplatelets (GNPs), ethanol (EtOH), methanol (MeOH), isopropanol, dioxane, toluene, and ethyl acetate, were purchased from Sigma Aldrich (Madrid, Spain). All commercial chemicals used in this study were of analytical reagent grade.

### 2.2. Extraction Process

Two ultrasonic methods were used to extract lignin from agricultural and forestry wastes.

#### 2.2.1. Sample Pre-Treatment

The waste samples (agricultural or forestry waste) were conditioned according to the procedure described by Moubarik et al. [35] to separate soluble hemicelluloses and increase lignin purity. This pre-treatment involved air drying the samples at room temperature to a moisture content of 8–10%. The samples were then crushed, ground, and sieved. A total of 10 g of the dry powder was washed with hot water at 80 °C for 2 h at a ratio of 1:20 *w*/*w*. After cooling to 25 °C, the residue was washed with fresh water at room temperature and centrifuged at 2000 rpm for 10 min. 

An additional and optional pre-treatment step involved washing the residue with 3.7% H_2_SO_4_ (1:20). This was performed first at 80 °C for 40 min in an ultrasonic bath and then at 90 °C for 23 min with mechanical stirring. 

#### 2.2.2. Method 1: Ultrasonic Bath Extraction of Black Liquor 

The ultrasonic bath extraction was performed using a Bandelin Sonorex. To obtain the black liquor, 250 mL of NaOH at various concentrations (0.5 M, 1 M, 2 M, and 5 M) was added to a 500 mL beaker containing 10 g of the pre-conditioned sample. Different NaOH concentrations were used to define the optimum conditions for lignin extraction with the minimum amount of NaOH. The mixture was placed in an ultrasonic bath equipped with a thermometer and a mechanical stirrer. The ultrasonic bath was operated for 4 h at 56 °C with stirring speed set to 1 and the power set to 0.5. The mixture was then filtered under vacuum, and the black liquor was stored in the refrigerator. The solid fractions (containing cellulose) were washed until neutral pH, dried overnight at 60 °C, and weighed. Lignin was precipitated by adding concentrated sulfuric acid or hydrochloric acid dropwise to 100 mL of black liquor until a pH of 2 was achieved. The mixture was shaken for 1 h, filtered, and the solid phase (lignin) was washed until the pH exceeded 5. Finally, the lignin was dried in an oven at 60 °C for 12 h and weighed.

#### 2.2.3. Method 2: Ultrasonic Probe Extraction of Black Liquor

The ultrasonic probe extraction was performed using a QSonica Sonicators ultrasonic probe. Similar to Method 1, 250 mL of NaOH at various concentrations (0.5 M, 1 M, 2 M, and 5 M) was added to a 500 mL beaker containing 10 g of the pre-conditioned sample. This mixture was heated on a hot plate to approximately 70–80 °C with continuous stirring. Once the temperature was reached, the ultrasonic probe was inserted, and a cycle was programmed with amplitude of 50 and a total duration of 1 h, with an active time of 10 min and a pause time of 5 min. After the reaction, the mixture was filtered under vacuum, and the procedure continued as in Method 1 to obtain cellulose, black liquor, and lignin.

The lignin yield was calculated using the following Equation (1):

(1) $$Yield\;lignin=\frac{weight\;of\;dried\;lignin\;(g)}{weight\;of\;dried\;waste\;(g)}\times 100,$$

#### 2.2.4. Determination of Residual Monomers (Building Blocks) after Lignin Precipitation

Following lignin extraction and precipitation, the residual black liquor was treated to analyze the building blocks liberated during lignin extraction. Two different analytical methods were applied: a gas chromatographic method for volatile and semi-volatile compounds, and a liquid chromatographic method for the non-volatiles. 

For the analysis of volatile and semi-volatile compounds, the procedure involved a double liquid–liquid extraction; 25 mL of the black liquor residue was mixed with 5 mL of toluene. The organic phase (top) was collected and stored after extraction. To extract other monomers not solubilized in toluene, 5 mL of ethyl acetate was added to the remaining 25 mL of the aqueous phase, and the organic phase was recovered again. In both cases, the organic phases were dried using anhydrous sodium sulfate to remove water, and 1 µL was injected into the gas chromatograph–mass spectrometer (GC/MS). The equipment consisted of a Shimadzu (Kyoto, Japan) Nexis GC-2030 coupled to an MGCMS-QP2020NX detector, equipped with a 25 m long, 0.32 mm ID, and 1.2 µm layer thickness capillary column (Agilent, CP-SIL 5 CB, Santa Clara, CA, USA). The GC injector port temperature was set at 280 °C and operated in split mode at a ratio of 10:1. Helium was used as the carrier gas at a flow rate of 1.25 mL/min. The oven temperature was initially set to 70 °C and held for 1 min, then ramped up at 5 °C/min to 250 °C, and finally at 10 °C/min to 280 °C, and held for 5 min. The mass spectrometer was set to full scan mode (30–1000 *m*/*z*) to record the mass spectral data. The ion source and interface temperatures were maintained at 280 °C and 300 °C, respectively. Peak identification was performed using characteristic mass spectral ions of the monomers, by means of comparison with the NIST database.

Furthermore, the black liquor was analyzed by high-performance liquid chromatography (HPLC) for the identification of the non-volatile aromatic monomers. The HPLC system was a Shimadzu Nexera 40 series, equipped with an autosampler, a quaternary pump (maximum pump pressure of 700 bar), and a DAD detector. The separation method was based on the method described by Fischer et al. [36]. A C18 column (Agilent Zorbax ODS, 250 × 4.6 mm, particle size 5 μm) and a linear gradient were used for separation, where component A was an aqueous solution of NaH_2_PO_4_ (pH 3.35), and component B was a mixture of acetonitrile, 0.03 M NaH_2_PO_4_ (pH 3.45) and methanol (60, 30, and 10%, respectively). The gradient has the following steps: (a) 0–4 min: linear gradient from 100% to 94% of A; (b) 4–10 min: linear gradient from 94% to 77.2% of A; (c) 10–18 min: linear gradient from 77.5% to 0% A. The flow rate used was 1.2 mL/min. Absorbance of the eluent was monitored at 280 nm. Standards of the most common phenolic lignin monomers were used to identify the observed peaks: vanillinic acid, syringic acid, vanillin, p-coumaric acid, syringaldehyde, acetovanillone, ferulic acid, acetosyringone, 2-methoxy-p-methylphenol, m-cresol, and catechol. A 40-fold dilution with mobile phase A was performed on black liquor samples before injection (10 µL).

#### 2.2.5. Purification and Characterization of Lignin

After lignin extraction, lignin purification was performed according to the method described by Liu et al. [24], which involves washing 3.37 g of lignin with 100 mL of THF for 1 h. After filtration, two fractions were obtained. The liquid fraction was transferred to a rotary evaporator to isolate the heavy oil. The insoluble fraction was dissolved in NaOH and then precipitated with HCl to obtain the purified lignin, insoluble at acidic pH.

FTIR spectroscopy was employed to analyze the lignin samples subjected to water and acid pre-washing treatments, as well as THF purification following the extraction process. The samples were pressed against the diamond crystal surface using a spring-loaded anvil of a Bruker Tensor 27 equipped with ZnSe lenses. Spectra were recorded within the absorption bands in the range of 600–4000 cm^−1^ in Attenuated Total Reflectance (ATR) mode, with a resolution of 4 cm^−1^ and 32 scans per sample. The assignment of the main absorption bands was based on [37]. Baseline corrections were applied to the FTIR spectra for accurate interpretation. 

### 2.3. Depolymerization of Lignin Obtained from River Cane, Rice Husks, Broccoli, Wheat Straw, and Olive Stones

#### 2.3.1. Lignin Depolymerization by High-Pressure Reactor

An ILSHIN Autoclave high-pressure laboratory reactor with a 500 mL reaction vessel and a 360 mL removable liner was used for lignin depolymerization. The equipment can handle temperatures from 25 to 350 °C and pressures up to 20 MPa. The methodology followed was based on the procedure described Liu et al. [1], with slight modifications, as they performed the depolymerization in a small-volume Parr reactor. A 250 mL round-bottom flask was filled with 100 mL of black liquor, which was then placed in a rotary evaporator to remove 50% of the solvent (water). Subsequently, 60 mL of ethanol was added. The mixture was then transferred to the reactor beaker and placed in the pressure reactor for 4 h at 250 °C at 5.9 MPa.

**Figure 1:**
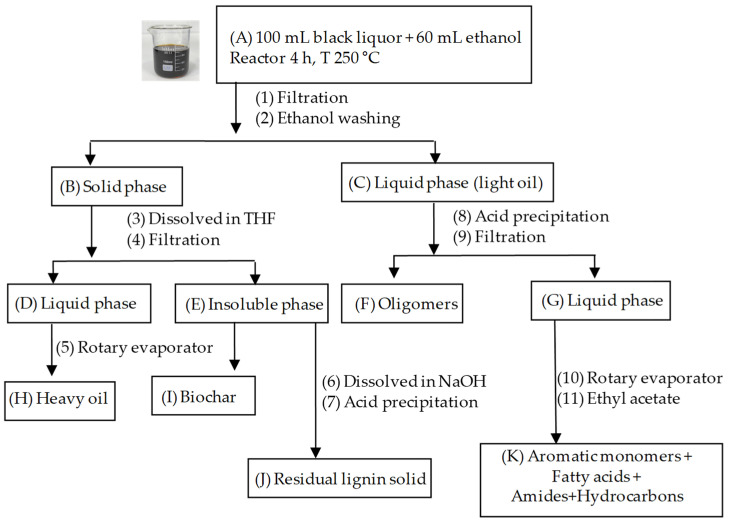
Scheme of lignin depolymerization in a pressure reactor and further processing to obtain aromatic monomers and other derivatives.

The extraction procedure used to separate and recover the different components after lignin depolymerization is shown in Figure 1. At the end of the depolymerization reaction (A), the mixture was filtered under vacuum (1) and washed with ethanol (2). The solid phase (B) was obtained, dissolved in 20 mL of THF (3), and filtered (4) to obtain a new liquid phase (D). The solvent was removed using a rotary evaporator (5), yielding a heavy oil (H). A portion of the insoluble phase (E) was dissolved in NaOH (6) and precipitated by the addition of HCl (7), resulting in lignin that could not be depolymerized (J). The portion of the insoluble phase that did not dissolve in NaOH was identified as biochar (I). The liquid phase consisting of a light oil (C) was treated with hydrochloric acid (8) and filtered (9) to obtain a solid phase (F) corresponding to the oligomers and a liquid phase (G). The latter was evaporated to dryness in a rotary evaporator (10) and redissolved in ethyl acetate (11). The residue (K) contained aromatic monomers and other derivatives.

A Heidolph rotary evaporator was used for solvent evaporation. It is equipped with a vacuum pump and contains a bath with a controllable temperature of up to 210 °C and a maximum rotational speed of 250 rpm.

#### 2.3.2. Lignin Depolymerization by Microwave

**Figure 2:**
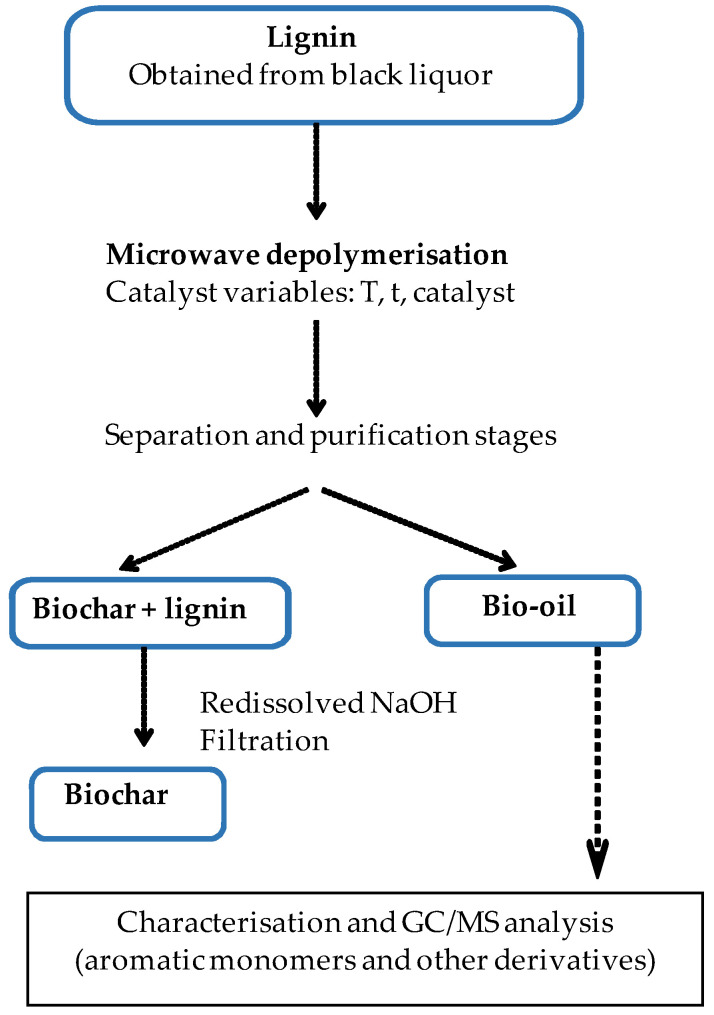
Scheme for microwave depolymerization of lignin.

Figure 2 shows the process of lignin depolymerization by microwaves. It was carried out using a Discover SP device from Vertex Technics S.L (Barcelona, Spain).

The procedure was as follows. A total of 25 mL of solvent (see below) was added to a 100 mL beaker containing 10 mg of GNPs. This mixture was sonicated for 15 min to condition the catalyst. Then, 1 g of lignin was added and the mixture was sonicated again for 10 min. Finally, it was placed into a microwave oven with the following settings: three temperatures (80, 120, and 160 °C), 250 psi of pressure, 200 W of power, two different reaction times (15 and 30 min), and four solvents used both pure and in mixtures (MeOH, H_2_O, isopropanol, dioxane, MeOH/H_2_O, MeOH/isopropanol, and isopropanol/H_2_O). The microwave oven parameters were controlled by the Sinergie software. After the reaction, the mixture was filtered. The solid phase, consisting of biochar and remaining lignin, was redissolved in 10 mL of NaOH (2 M). The mixture was filtered again to recover the biochar. The liquid phase obtained from the microwave depolymerization process, consisting mainly of bio-oil, was transferred to a round-bottom flask where the solvent was completely evaporated using a rotary evaporator. The residue was redissolved in ethyl acetate (25 mL). To remove impurities, a liquid–liquid extraction was performed with an aqueous solution of HCl (pH 1). The organic phase (ethyl acetate) was separated and treated with anhydrous sodium sulfate to remove residual water. It was filtered to remove the sodium sulfate, the ethyl acetate was evaporated by rotary evaporation, and finally, the residue was redissolved in the minimum amount of THF for GC-MS analysis. For HPLC analysis, a solvent change was carried out. A total of 0.5 mL of the solvent was evaporated and the obtained residue was redissolved in methanol and diluted, as described in Section 2.2.4.

#### 2.3.3. Lignin Depolymerization by Ultrasonic Bath or Probe

The method used for depolymerizing lignin by ultrasonic bath or probe is the same as that described in the extraction procedure with slight modifications. In both cases, 0.5 g of lignin and 60 mL of ethanol were added to a 250 mL beaker. For lignin depolymerization by ultrasonic bath, the bath conditions were (P/80/320 W, F: 35 kHz). The reaction was carried out for 4 h at a bath temperature of approximately 56 °C. For lignin depolymerization by ultrasonic probe, using a QSonica ultrasonic processor, model CL334, the reaction was performed for 1.5 h with an amplitude of 50, and a cycle of 10 min of ultrasonic activity followed by 5 min of rest, making the effective duration of the ultrasonic treatment 1 h and the temperature of the system about 60 °C. In both cases, the sample was filtered and treated as described in Figure 1 after the depolymerization reaction.

## 3. Results

### 3.1. Extraction Process

#### 3.1.1. Lignin Extraction Process with Ultrasonic Bath or Probe

In order to optimize the extraction method, a study with the same residue was performed. Table 1 presents the results obtained for the extraction of lignin from river cane using an ultrasonic bath (Method 1, described in Section 2.2.2). The studies were conducted by adding 10 g of pre-conditioned waste to 250 mL of NaOH at different concentrations, at 50 °C, varying the reaction time and the acid used for precipitation, in triplicate. In all cases, HCl or H_2_SO_4_ was added dropwise until a pH of 2 (necessary for lignin precipitation) was reached. The lignin yield was determined after precipitation, washing, and drying at 60 °C, using the method described previously [38].

The first section of Table 1 shows that, under constant reaction time conditions, the lignin extraction yield increases with higher NaOH concentrations. However, at a concentration of 5 M NaOH, a decrease in extraction yield is observed. The increase in pH produces a higher amount of sodium hydroxide precipitation, as a consequence of which the lignin has to be washed more times, resulting in a higher loss of lignin during the washings and therefore a lower percentage of lignin obtained. The second section of Table 1 indicates that reaction time has no significant effect on the yield. Additionally, a higher yield is obtained with HCl compared to H_2_SO_4_ when precipitating lignin from black liquor with two different acids. Using sulfuric acid results in more salts, similar to the 5 M NaOH concentration, requiring more water for washing and thus reducing yield. The optimal result from the ultrasonic bath was a 28.21% yield with 2 M NaOH for 4 h, with 2.5–2.6 mL of concentrated acid per 50 mL of black liquor.

**Figure 3:**
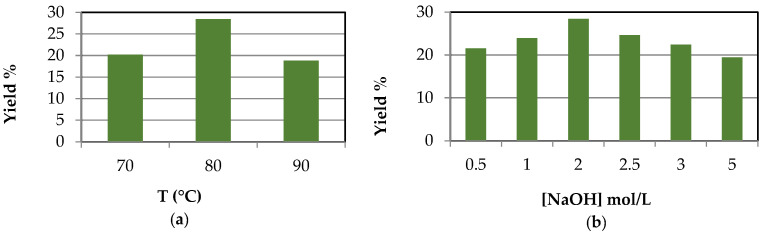
Lignin extraction by ultrasonic probe, V_NaOH_ 250 mL, lignin mass 10 g. (**a**) Different temperatures, [NaOH] 2 M. (**b**) Different [NaOH], T 80 °C.

The results for lignin extraction using the ultrasonic probe method are illustrated in Figure 3. Two studies were conducted on the extraction of lignin from river cane using an ultrasonic probe. Figure 3a shows the extraction with 2 M NaOH at varying temperatures, with the best results at 80 °C. Increasing the temperature above 80 °C reduced the yield. Figure 3b demonstrates lignin extraction with increasing NaOH concentrations at 80 °C, with the best results at 2 M NaOH, due to higher concentrations producing more salts, contaminating the lignin, and requiring further washing and purification and, as a result, a loss of yield.

**Figure 4:**
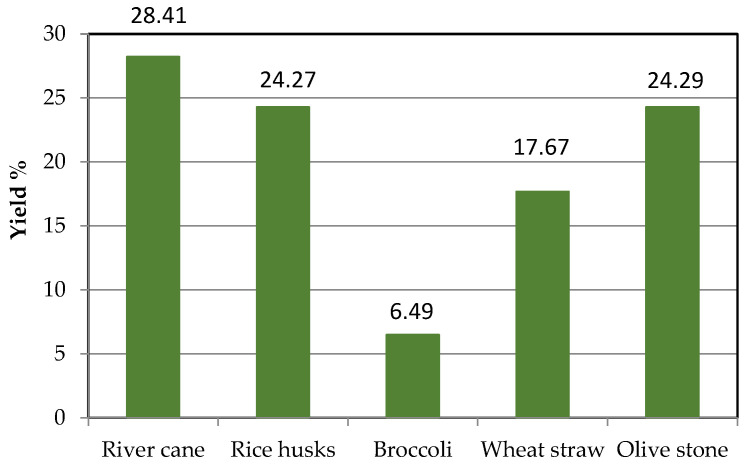
Lignin extraction yields from various wastes.

Figure 4 shows the lignin extraction yields from different types of residues using the ultrasonic probe. The conditions were 2 M NaOH at 80 °C. The results obtained align with literature values of lignin content in the studied products, indicating the method’s efficiency; this lignin is washed, not purified. Table 2 shows the composition of the five wastes analyzed in this study. These percentages of lignin, hemicellulose, and cellulose were obtained from different literature articles [39,40,41,42,43,44,45,46]

#### 3.1.2. Purification and Characterization of Lignin

**Figure 5:**
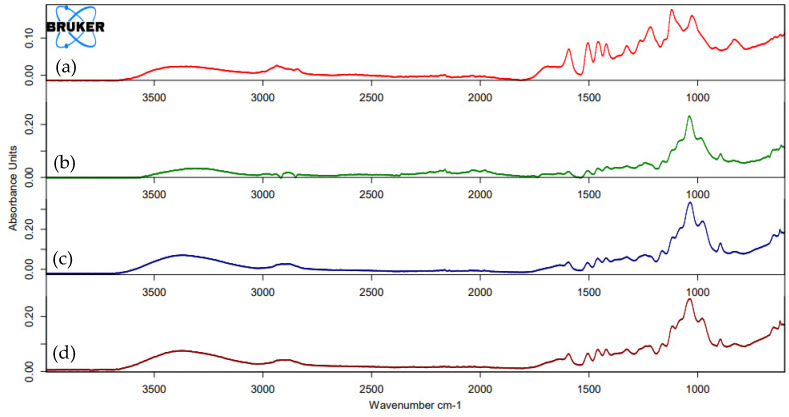
IR spectra of (**a**) unpurified lignin, (**b**) THF purified lignin, (**c**) H_2_O pre−washed lignin, (**d**) H_2_SO_4_ pre−washed lignin. All prepared from river cane extracts with the probe method.

The IR spectra of the lignin samples are shown in Figure 5. The signal at 1730 cm^−1^ corresponds to carbonyl (C=O) group stretching in non-conjugated ketones, carbonyl, and aliphatic groups present in all wood components, mainly hemicellulose [47,48]. Peaks around 1400 cm^−1^ are associated with ring-stretching modes strongly coupled to C-H in-plane deformation, and peaks at 1200 cm^−1^ are associated with the guaiacyl structure in lignin molecules. Some of these peaks can also be related with the presence of carbohydrate interferences, so the different purification methods applied are evaluated by means of the comparison of the intensity of strategic peaks [49]. Around 1700 cm^−1^ is assigned to hemicellulose; peaks around 1350 and 1150 cm^−1^ are attributed to polysaccharides.

As Figure 5 shows, pre-washing the samples before lignin extraction produces a cleaner spectrum, decreasing desired peak intensities. However, no significant differences are detected when the second step of pre-washing with sulfuric acid is applied. The same results were obtained with the purification of lignin with THF. Therefore, a single-step pre-washing method with hot water was finally applied for the rest of the study.

A comparison of the IR spectra of lignin obtained from river cane, rice husks, broccoli, wheat straw, and olive stones shows similar results. Table 3 lists the wavelength values for different functional groups for the different lignins obtained from each residue.

Comparing the spectra with [37], peaks at 1594–1510 cm^−1^ can be attributed to aromatic skeletal vibrations. Bands at 1459 cm^−1^ are associated with methyl and methylene C-H bending groups. Peaks at 1426–1424 cm^−1^ are related to ring stretching modes with C-H plane deformation. Peaks between 1367 and 1364 cm^−1^ indicate C-O stretching of guaiacyl units, and 1327–1345 cm^−1^ peaks are associated with C-O stretching of guaiacyl/syringyl structures in lignin molecules. Peaks at 1242–1213 cm^−1^ are associated with the guaiacyl structure, and 1044–1030 cm^−1^ peaks are associated with C-O stretching of primary alcohols. Bands at 914–813 cm^−1^ are attributed to C-H bending of guaiacyl and syringyl units, with the band at 854 cm^−1^ being typical of the aromatic ring structure of guaiacyl.

#### 3.1.3. Determination of Residual Monomers 

After lignin precipitation, the remaining black liquor was subjected to a liquid–liquid extraction (LLE) in order to analyze potential aromatic monomers or other low-molecular-weight compounds of interest. A double LLE was performed with toluene and ethyl acetate. The extracts from the black liquor residue were analyzed by gas chromatography–mass spectrometry (GC-MS). Various building blocks were identified, although this is a non-quantitative method. Short-chain hydrocarbons with retention times under 5 min were obtained in all the analyzed extracts, forming an unresolved complex mixture or hump difficult to identify. Table 4 lists the monomers obtained from different residues, highlighting those aromatic monomers suitable as building blocks.

Figures S1–S4 show extracts from river cane, rice husk, broccoli, and wheat straw in toluene and ethyl acetate, revealing phenol-derived monomers and lignin derivatives with retention times under 5 min. For river cane, components such as guaiacol, acetovanillone, acetosyringone, 2-2′methylenebis(4-methyl-6-tert-butylphenol), and other monomers such as oleamide, stereamide, and palmitic acid were obtained in toluene extracts. In ethyl acetate extracts, lauric acid, L-ascorbyl dipalmitate, fatty acid mixtures, and 2,2′-methylenebis(4-methyl-6-tert-butylphenol) were found. For rice husk, aromatic monomers included vanillin, 3-(3-hydroxyphenyl)-2-propenoic acid, 4-hydroxy-2-methoxybenzaldehyde, fatty acids, and also 2-2′methylene-bis(4-methyl-6-tert-butylphenol). The coumarin production is remarkable; this monomer is used as an anticoagulant for the treatment of excessive clotting disorders and for certain heart conditions. For the extraction of lignin from broccoli, apart from short-chain hydrocarbons, only palmitic acid was obtained. Wheat straw yielded the highest amount of aromatic monomers derived from lignin such as acetovanillone, syringaldehyde, acetosyringone, 4-vinylguaiacol, 4-hydroxy-2-methoxy-benzaldehyde, and 2-2′-methylenebys(4-methyl-6-tert-butylphenol). Oleamide can be used to give a smooth surface finish to PE films or PP articles; this grade tends to have a short-term effect and is used to prevent PE films from sticking.

**Figure 6:**
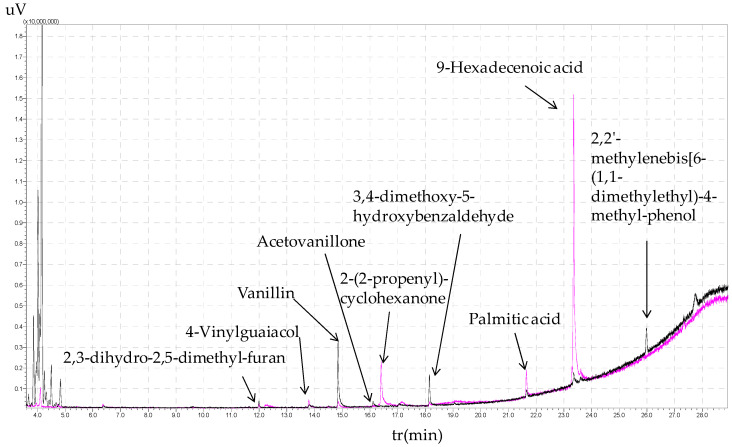
Mass spectra obtained from GC/MS chromatography for the residues of black liquor extracted from olive stones (toluene extract: black line; ethyl acetate: pink line).

Olive stone extracts (Figure 6) from the residue of black liquor in toluene and ethyl acetate contained aromatic monomers and lignin derivatives, including 2,3-dihydro-2,5-dimethyl-furan, vanillin, acetovanillone, 3,4-dimethoxy-5-hydorxybenzaldehyde, 4-vinylguaiacol, 2-(2-propenyl)-cyclohexanone, 2,2′-methylenebis [6-(1,1-dimethylethyl)-4-methyl-phenol, and palmitic acid (9-hexadecenoic acid) in larger quantities in ethyl acetate.

After the extraction, a high-performance chromatography study was also carried out to determine the aromatic derivatives and other lignin derivatives, especially those that were not volatile and could not be obtained by gas chromatography. The HPLC chromatograms obtained for the standards of different phenolic lignin monomers and for the black liquor of rice husks and black liquor of river cane were as shown in Figure S5. As can be seen in the chromatograms, p-coumaric acid, ferulic acid, and vanillin were detected for rice husk black liquor, and p-coumaric acid and ferulic acid for river cane black liquor. The same peaks were detected in the rest of samples, obtaining higher intensities for rice husk.

### 3.2. Depolymerization of Lignin

Lignin depolymerization was carried out using a high-pressure reactor, microwave, and ultrasonic bath or probe. In all cases, lignin was purified post-extraction. The results are shown in Table 5, Table 6, Table 7 and Table 8.

#### 3.2.1. Depolymerization in High-Pressure Reactor

The aim was to depolymerize lignin under severe conditions in a high-pressure reactor. In this case, the amount of lignin obtained for the different wastes in 100 mL of black liquor is not the same. These amounts of lignin have been determined on the basis of the results obtained previously by lignin extraction (Section 3.1.1). The amount of oligomers for wheat straw is higher than that obtained for river cane and rice husk, but this value is even higher for broccoli, which may be due to the fact that cruciferous plants have more flavonoid groups, i.e., larger phenolic compounds with antioxidant capacity, whose bonds are more difficult to break to obtain monomers. Consequently, together with wheat straw, broccoli has the lowest number of aromatic monomers. It is also interesting to note that for olive stones, the percentage of heavy oil is higher, but the percentage of biochar is lower.

**Figure 7:**
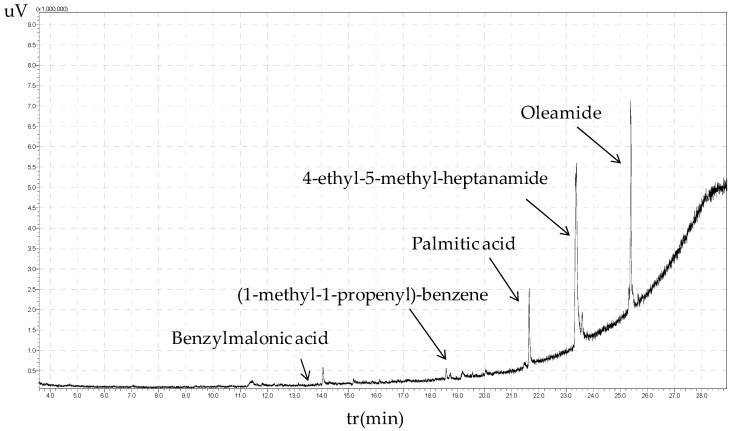
GC/MS chromatogram for lignin depolymerization of olive stones with high−pressure reactor, ethyl acetate extract.

Figure 7 shows a GC-MS chromatogram for lignin depolymerization of olive stones extracted with ethyl acetate, revealing aromatic monomers such as benzylmalonic acid, (1-methyl-1-propenyl)-benzene, palmitic acid, 4-ethyl-5-methyl-heptanamide, and oleamide. Most lignin derivatives were fatty acids or their amides.

#### 3.2.2. Lignin Depolymerization in Microwave

Firstly, microwave depolymerization was carried out on cane lignin and the results of this depolymerization are shown in Table 6. This study was carried out with and without graphene nanoplatelets (GNPs) at different temperatures and times, for the same solvent or solvent mixture. The use of GNPs as substrates is due to the fact that they are materials with a typical thickness of about 8 nm, available in different sizes and consisting of small sheets formed by small graphene sheets in the form of platelets, similar to those present in the walls of nanotubes. In all cases, an increase in temperature and time leads to an increase in depolymerization. In addition, the use of the GNP catalyst increases the percentage of depolymerization and aromatic monomers.

The best results were obtained with dioxane as solvent, achieving an 80–90% depolymerization yield at 120 °C with GNPs, and 71–73% aromatic monomers with isopropanol/H_2_O and 60–77% with methanol/H_2_O mixtures at 120 °C.

Figure S6 presents the GC-MS chromatogram for the depolymerization of river cane lignin in methanol, identifying monolignols such as guaiacol, vanillin, cinnamic acid, and acetovanillone. The application of graphene nanoplatelets (GNPs) in microwave-assisted depolymerization has led to a patent (ES 2 961 134). The results of this study indicate that microwave technology for lignin depolymerization is constrained by the nature of the involved compounds, impacting the efficiency of microwave radiation transfer into heat. Consequently, there is a necessity for lignin depolymerization methods that minimize reaction time and temperature, while also reducing reliance on costly and environmentally harmful catalysts. The incorporation of GNPs addresses these challenges by providing an efficient method for lignin depolymerization using carbonaceous microwave radiation. GNPs, as carbon-based nanomaterials, serve as effective radiation susceptors and depolymerization catalysts. Specifically, GNPs facilitate the microwave-assisted depolymerization of lignin into molecules with high technological value, such as vanillin and various phenolic derivatives. Due to their structure, GNPs can be functionalized through covalent bonds or π-π stacking interactions with the phenolic groups of lignin. Furthermore, their simple graphitic structure makes GNPs excellent electrical and thermal conductors, significantly enhancing microwave radiation absorption and improving lignin depolymerization efficiency.

Regarding the HPLC analysis, Figure S7 shows, as an example, the chromatogram obtained for the aromatic compounds derived from river cane lignin after microwave depolymerization. Ferulic acid, vanillin, and acetovanillone were identified. Similar results were observed for the rest of the samples.

A comparison was then made with the microwave depolymerization of the lignin obtained with the different wastes, and the results are shown in Table 7. This study was carried out to check whether the microwave depolymerization with the best conditions obtained for river cane lignin (1 g lignin, solvent/dioxane, 120 °C, and 15 min) was also suitable for the other wastes.

The findings reveal that GNP-catalyzed depolymerization consistently enhances lignin breakdown and aromatic monomer extraction. Among the tested lignin sources, river cane lignin exhibits the highest depolymerization efficiency, followed by olive stones, rice husks, broccoli, and wheat straw lignins, in descending order of performance. This trend corresponds to the decreasing percentage of unreacted lignin, with wheat straw showing the highest amount, followed by broccoli, rice husks, olive stones, and river cane. Biochar production was most significant for rice husk lignin, followed by broccoli, wheat straw, olive stones, and river cane. Chromatograms in Figure S8 illustrate the separation of aromatic derivatives from other lignin breakdown products. The ethyl acetate extracts from all depolymerizations contained aromatic monomers and lignin derivatives, along with butylated hydroxytoluene, fatty acids (palmitic and linolenic acid), and amide derivatives (tetradecamide, oleamide, and stearamide).

#### 3.2.3. Depolymerization of River Cane by Different Methods: Ultrasonic Bath or Probe, High-Pressure Reactor, and Microwave Depolymerization

A comparison was made between the results obtained with the ultrasonic probe and bath, high-pressure reactor, and microwave (Table 8). The microwave method yielded the highest depolymerization percentage, aligning with the primary objective of this study. Additionally, microwave depolymerization required less reaction time, resulting in lower energy consumption, making it more cost-effective and environmentally friendly. Ultrasonic depolymerization achieved a 78.5% depolymerization rate, whereas reactor-based depolymerization produced substantial biochar due to the high temperatures, converting some lignin and aromatic monomers into biochar. In contrast, microwave depolymerization yielded a high depolymerization rate of (90.89%) with minimal biochar formation (6.43%), and no detectable oligomers or heavy oil.

## 4. Discussion

This study explores various lignin extraction and depolymerization methods that have been investigated and developed to obtain valuable compounds (building blocks) from five different agricultural and forestry wastes: river cane, rice husks, broccoli, wheat straw, and olive stone. The residues were selected according to the strategies of the Agromatter project, which aims for the revalorization of local wastes that are typically incinerated. In addition to the aim of this work, the Agromatter project proposes other ways to valorize these types of by-products, such as the extraction of cellulose, and utilize it to produce cellulose nano-reinforcements for food packaging or combine cellulose with nonwoven materials to create infused panels for acoustic and thermal insulation (activities performed by other partners of the project).

Regarding the extraction of lignin, two different ultrasound-assisted extraction methods have been evaluated (probe and bath). The findings indicate that the optimal method is the probe method, significantly reducing extraction time compared to the ultrasonic bath method. During lignin extraction, hemicellulose and other polysaccharides coprecipitate with lignin, as confirmed by IR spectroscopy. Thus, some purification methods were evaluated, like pre-washing steps and purification of the obtained lignin. While the best results were obtained using THF for lignin purification, this solvent is not environmentally friendly. Nevertheless, simple pre-washing of the agricultural residues also produced similar results on the lignin purity quality. Finally, the optimal conditions for lignin extraction were identified as using 2 M NaOH at 80 °C with an ultrasonic probe, after prewashing of the waste sample with hot water. The extraction yields from different residues were as follows: river cane (28.21%), rice husks (24.27%), broccoli (6.48%), wheat straw (17.66%), and olive stones (24.29%). These results align well with the lignin content reported in the literature, demonstrating the effectiveness of the ultrasonic probe method. The IR spectra of the extracted lignins indicated the presence of typical lignin functional groups, confirming the successful isolation of lignin.

These processes are industrially scalable. Comparing this method with industrial soda lignin extraction methods, this method avoids the use of high-pressure systems and reduces operating time and temperature satisfactorily. This results in lower energy and economic costs, which is beneficial from an environmental perspective.

The sonomechanical energy of ultrasound promotes the disintegration of solute particles and enhances solvent accessibility by increasing the surface area of the reactants. Acoustic energy associated with ultrasonic can also initiate or enhance chemical reactions, leading to the formation of free radicals and other reactive species, which further drive depolymerization processes. A GC-MS and HPLC screening analysis was therefore carried out to identify the aromatic monomers released during lignin extraction. Several aromatic monomers were identified, indicating that some depolymerization processes occur simultaneously with extraction. Some of the identified monomers were vanillin, p-coumaric acid, ferulic, and guaiacol.

Focusing on the depolymerization processes, the microwave-assisted depolymerization yielded the highest aromatic monomer production, achieving a total depolymerization percentage of 90.89%. In the microwave depolymerization of river cane lignin, the primary aromatic monomers obtained were guaiacol, vanillin, cinnamic acid, and acetovanillone. The use of catalysts in microwave depolymerization is often costly; however, this issue is mitigated by employing graphene nanoplatelets (GNPs). GNPs significantly enhanced the efficiency of the microwave depolymerization process due to their excellent thermal and electrical conductivity. The use of GNPs resulted in higher depolymerization rates and a greater yield of aromatic monomers compared to reactions without the catalyst. The ultrasonic probe method also showed promising results with a depolymerization rate of 78.5%, while the high-pressure reactor method was less efficient, primarily producing biochar due to the high temperatures involved. The results of the microwave depolymerization of lignin from different residues are summarized in Table 7. River cane lignin showed the highest depolymerization efficiency, followed by olive stones, rice husks, broccoli, and wheat straw. The high depolymerization efficiency and lower biochar production highlight the potential of microwave-assisted depolymerization as a viable industrial process. Regarding the monomer fraction, three main components were identified: ferulic acid, vanillin, and acetovanillone.

## 5. Conclusions

This study demonstrates the potential valorization of five agricultural and forestry wastes (river cane, rice husks, broccoli stems, wheat straw, and olive stone) for the production of high-value materials. The optimized ultrasonic probe method for lignin extraction used 2 M NaOH at 80 °C with an ultrasonic probe, achieving a yield of 28.41% for river cane. The microwave-assisted depolymerization with GNPs as a catalyst was the most effective, with depolymerization rates up to 90.89%. These findings contribute to the development of environmentally friendly methods for lignin valorization, supporting the principles of the circular economy. Future work will focus on two routes: (a) direct application of the obtained lignin into biodegradable plastics such as polylactic acid (PLA) produced by another research center within the consortium (Agromatter project); (b) application of the depolymerized lignin by means of separation of aromatic monomers using chromatography and addition to PLA for use in biodegradable containers and packaging. Beyond the scope of this project, lignin is a valuable biobased feedstock. Lignin polymers find direct use in the production of lignosulfonates, carbon materials, polymer resins, adhesives, and as a copolymer or additive in new materials and composites. In addition, lignin building blocks are a potential source of high-value aromatic compounds in the petrochemical industry, with applications in such diverse sectors as polymer, energy, or pharmaceutical industries. Thus, lignin is considered as a renewable and affordable solution to reduce our dependence on fossil resources.

## 6. Patents

Publication number: ES 2 961 134 (Método de despolimerización de Lignina mediante microondas).

## Figures and Tables

**Table 1:** Results for lignin extraction from river cane using the ultrasonic bath method.

[NaOH] (M)	t_US_ (h)	Yield (%)	Acid
0.5	4	25.12	HCl
1	4	26.15	HCl
2	4	28.21	HCl
5	4	15.43	HCl
2.5	1.5	22.59	HCl
2.5	2	23.20	HCl
2.5	3.0	23.42	HCl
2.5	4.5	22.77	HCl
2	2	22.48	H_2_SO_4_
2	2	24.38	HCl

**Table 2:** Lignocellulosic composition of wastes (%).

Waste	Lignin	Cellulose	Hemicellulose
River Cane	22.4–29	24–43.1	22–26.8
Rice Husks	25–30	50	15–20
Broccoli	1.5–16	16	7.3–10.7
Wheat straw	15–20	29–38	24.6–39.1
Olive stones	26.9	32.1	34.8

**Table 3:** Infrared frequency signals.

Lignin	ν_vibra_	ν_C-H methyl/methylene_	ν_C-H aromatic_	ν_C=O guaiacyl/syringyl_	ν _guaiacyl_	ν_C-O alcohols_	ν_C-H guaiacyl/syringyl_
River cane	1594/1505	1459	1420	1327	1242	1035	896/824
Rice husk	1595/1507	1457	1419	1338	1261/1213	1035	879
Broccoli	1592/1504	1457	1420	1325	1217	1026	918/831
Wheat straw	1596/1508	1460	1420	1345	1245	1035	895
Olive Stones	1592/1511	1451	1423	1345	1246	1030	876/853

**Table 4:** Lignin extraction aromatic monomers and other derivatives.

	River Cane	Rice Husk	Broccoli	Wheat Straw	Olive Stones
Vanillin	-	x	-	-	x
Guaiacol	x	-	-	-	-
Acetovanillone	x	-	-	x	x
Syringaldehyde	-	-	-	x	-
Acetosyringone	x	-	-	x	-
Phenol	-	-	-	-	x
4-Vinylguaiacol	-	-	-	x	x
Coumarin	-	x	-	-	-
3-(3-hydroxiphenyl)-2-propenoic acid	-	x	-	-	-
2,3-dihydro-2,5-dimethyl-furan	-	-	-	-	x
4-hydroxy-2-methoxybenzaldehyde	-	x	-	x	-
2-(2-propenyl)-cyclohexanone	-	-	-	-	x
3,4-dimethoxy-5-hydroxybenzaldehyde	-	-	-	-	x
2-2′-methylenebys(6-(1,1-dimethylethyl)-4-methylphenol)	-	-	-	-	x
2-2′-methylenebys(4-methyl-6-tert-butylphenol)	x	x	-	x	-
Palmitelaidic acid	x	-	-	-	x
Palmitic acid	x	-	x	-	x
Etereamide/Oleamide	x	-	-	x	-

**Table 5:** Depolymerization of waste in a high-pressure reactor.

Sample	Lignin * (g)	Oligomers (%)	Heavy Oil (%)	Aromatic Monomers + Other Derivatives (%)	Biochar (%)
River cane	1.148	9.49	0.59	69.71	20.20
Rice husks	0.971	5.82	1.81	75.71	10.68
Broccoli	0.538	42.32	1.86	30.24	25.58
Wheat straw	0.707	16.81	1.81	31.09	50.28
Olive stones	0.972	16.65	3.93	58.88	18.52

* calculated from the results of lignin extraction obtained in 100 mL of black liquor for each residue.

**Table 6:** Lignin depolymerization of river cane with microwave.

Solvent	GNP_S_	T (°C)	t (min)	Depolymerized (%)	Aromatic Monomers (%)	Biochar + Lignin (%)
MeOH	0.01	80	15	55.41	9.91	31.35
MeOH	0.01	120	15	61.51	9.29	32.37
MeOH	0	120	15	58.12	6.38	32.75
MeOH	0.01	80	30	57.21	9.76	31.06
MeOH	0	80	15	49.66	7.22	26.28
MeOH	0.01	120	30	53.49	9.43	28.19
MeOH	0.01	160	15	58.93	17.44	32.25
MeOH	0	160	15	48.70	8.60	28.43
H_2_O	0.01	80	15	11.25	2.58	84.82
H_2_O	0.01	120	15	24.88	9.07	83.43
H_2_O	0	120	15	16.92	6.13	80.61
MeOH/H_2_O	0.01	80	15	43.27	17.26	58.78
MeOH/H_2_O	0.01	120	15	73.73	16.13	19.50
MeOH/H_2_O	0	120	15	71.90	7.53	27.36
MeOH/Isopropanol	0.01	80	15	33.93	18.88	73.62
MeOH/Isopropanol	0.01	120	15	42.54	21.44	73.85
MeOH/Isopropanol	0	120	15	36.64	20.51	67.68
Isopropanol	0.01	80	15	12.90	10.38	84.00
Isopropanol	0.01	120	15	17.56	12.85	82.74
Isopropanol	0	120	15	15.82	12.20	75.98
Isopropanol/H_2_O	0.01	80	15	57.36	4.97	4.62
Isopropanol/H_2_O	0.01	120	15	77.70	10.82	15.12
Isopropanol/H_2_O	0	120	15	60.31	10.63	11.14
Dioxane	0.01	80	15	79.90	22.99	15.64
Dioxane	0.01	120	15	90.89	54.87	9.97
Dioxane	0	120	15	85.29	25.73	17.58

**Table 7:** Microwave depolymerization of lignin for different wastes.

	GNP_S_ (g)	Depolymerized (%)	Aromatic Monomers (%)	Biochar (%)	Unreacted Lignin (%)
River cane	0	85.29	25.73	9.23	8.35
River cane	0.01	90.89	54.87	6.43	3.54
Rice husk	0	12.83	6.13	52.94	36.23
Rice husk	0.01	21.62	9.45	57.17	22.15
Broccoli	0	10.82	3.73	34.99	55.24
Broccoli	0.01	19.65	7.73	42.49	37.56
Wheat straw	0	9.23	4.93	23.18	67.15
Wheat straw	0.01	17.65	6.30	38.12	45.23
Olive Stones	0	35.61	10.30	29.12	36.23
Olive Stones	0.01	43.27	14.53	30.68	24.75

**Table 8:** Results of lignin depolymerization of river cane lignin with different methods.

River Cane Lignin	t (h)	Oligomers (%)	Heavy Oil (%)	Depolymerization (%)	Biochar (%)
Ultrasonic bath	4	0	7.52	22.64	0
Ultrasonic probe	1.5	2.08	2.04	78.50	17.38
High-pressure reactor	6	26.03	3.40	59.67	20.91
Microwave (dioxane)	0.25	-	-	90.89	6.43

## Data Availability

Data are contained within the article and the Supplementary Materials.

## Supplementary Materials

The following supporting information can be downloaded at: https://www.mdpi.com/article/10.3390/polym16141981/s1; Figure S1. Mass spectra obtained from GC/MS chromatography for the residue of Black Liquor extracted from river cane (toluene extract: black line; ethyl acetate: pink line); Figure S2. Mass spectra obtained from GC/MS chromatography for the residue of Black Liquor extracted from rice husk (toluene extract: black line; ethyl acetate: pink line); Figure S3. Mass spectra obtained from GC/MS chromatography for the residue of Black Liquor extracted from Broccoli (toluene extract: black line; ethyl acetate: pink line); Figure S4. Mass spectra obtained from GC/MS chromatography for the residue of Black Liquor extracted from Rice straw (toluene extract: black line; ethyl acetate: pink line); Figure S5. HPLC chromatograms: (A) Standards of different phenolic lignin monomers, (B) black liquor of rice husk and (C) black liquor of river cane; Figure S6. GC/MS chromatogram for lignin depolymerization of river cane with microwave in methanol, (toluene extract); Figure S7. HPLC chromatograms: for microwave depolymerized river cane lignin; Figure S8. GC/MS chromatogram for lignin depolymerization of other waste lignin with microwave, (ethyl acetate extract). Rice husk (blue line), broccoli (pink line), wheat straw (brown line) and olive stones (black line).
