# Supplementary material for: Extraction and Depolymerization of Lignin from Different Agricultural and Forestry Wastes to Obtain Building Blocks in a Circular Economy Framework
Source: Polymers (Basel). 2024 Jul 11;16(14):1981. doi: 10.3390/polym16141981 (PMC11280865; doi:10.3390/polym16141981)
Supplement: Supplementary file 1 [file polymers-16-01981-s001.zip › polymers-3065476-supplementary.pdf]

Supplementary Materials: The following supporting information can be downloaded at: www.mdpi.com/xxx/s1.

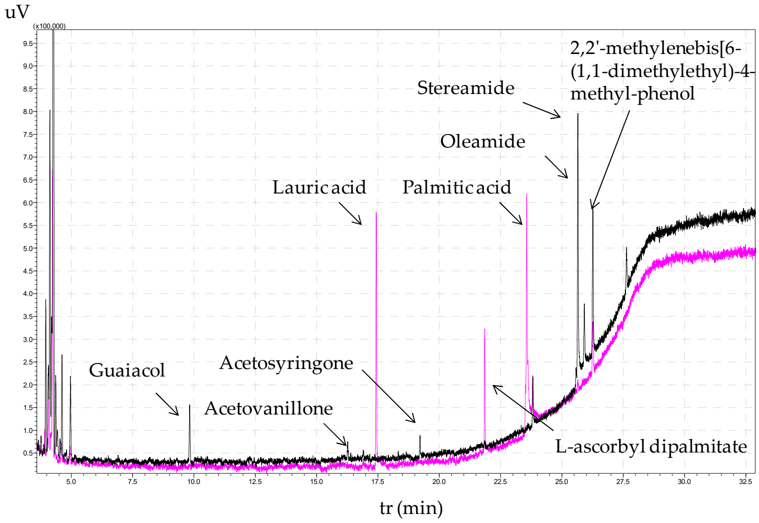

Figure S1. Mass spectra obtained from GC/MS chromatography for the residue of Black Liquor extracted from river cane (toluene extract: black line; ethyl acetate: pink line).

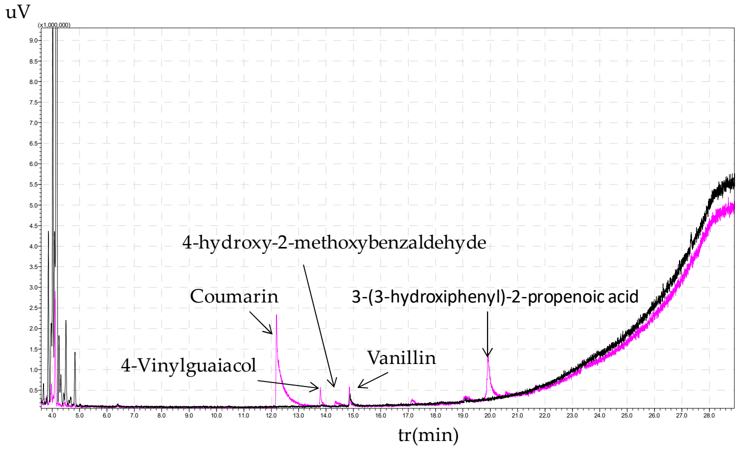

Figure S2. Mass spectra obtained from GC/MS chromatography for the residue of Black Liquor extracted from rice husk (toluene extract: black line; ethyl acetate: pink line).

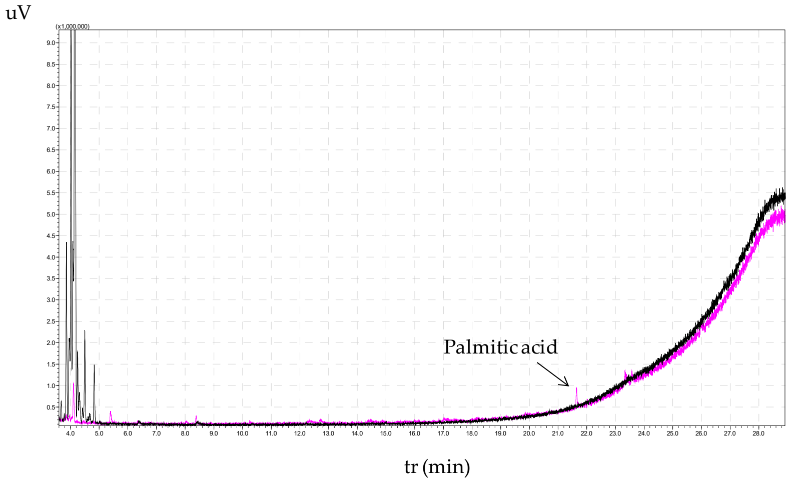

Figure S3. Mass spectra obtained from GC/MS chromatography for the residue of Black Liquor extracted from Broccoli (toluene extract: black line; ethyl acetate: pink line).

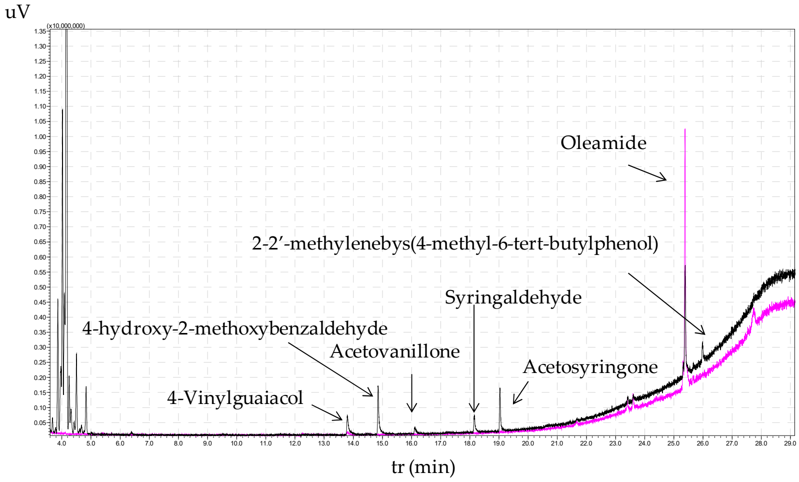

Figure S4. Mass spectra obtained from GC/MS chromatography for the residue of Black Liquor extracted from Rice straw (toluene extract: black line; ethyl acetate: pink line).

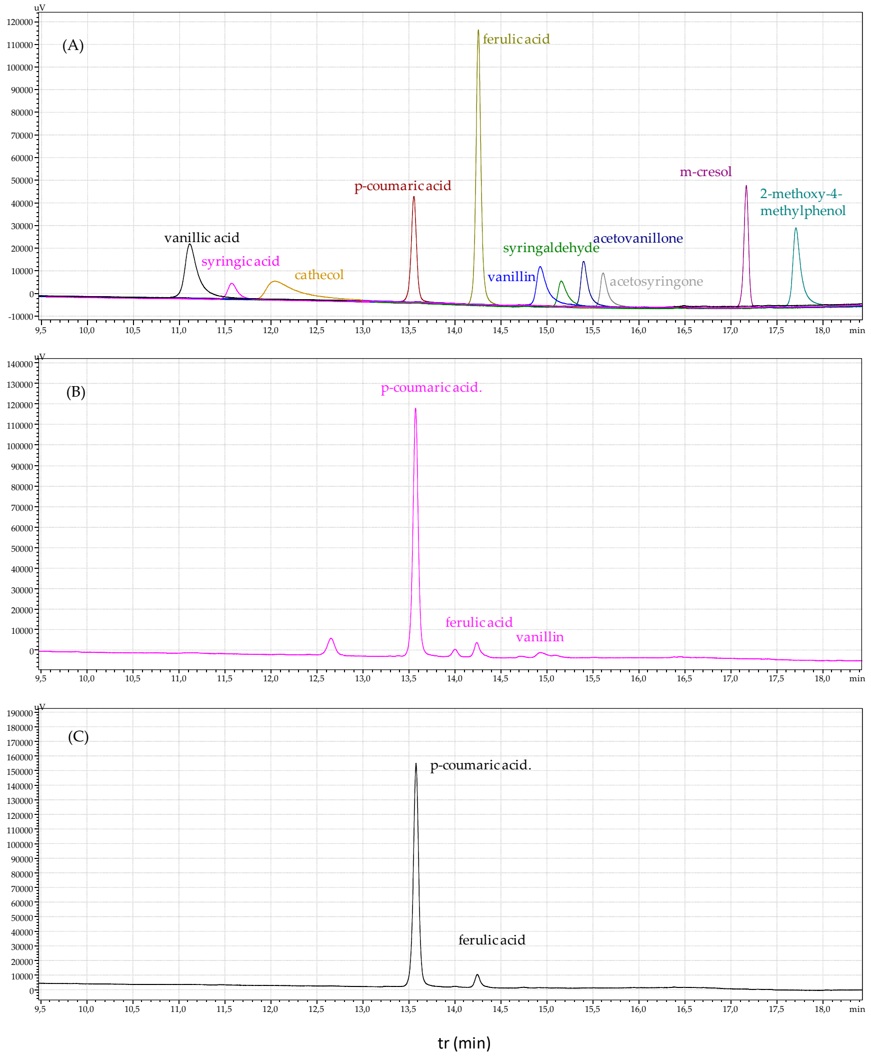

Figure S5. HPLC chromatograms: (A) Standards of different phenolic lignin monomers, (B) black liquor of rice husk and (C) black liquor of river cane.

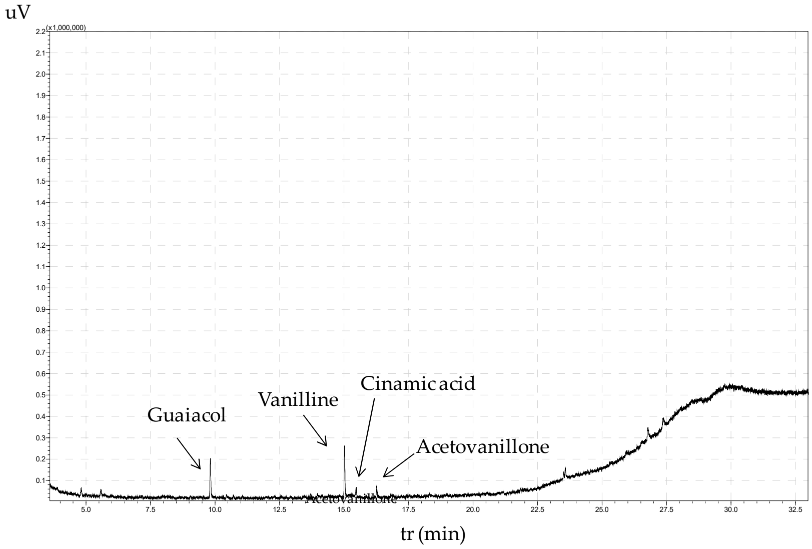

Figure S6. GC/MS chromatogram for lignin depolymerization of river cane with microwave in methanol, (toluene extract).

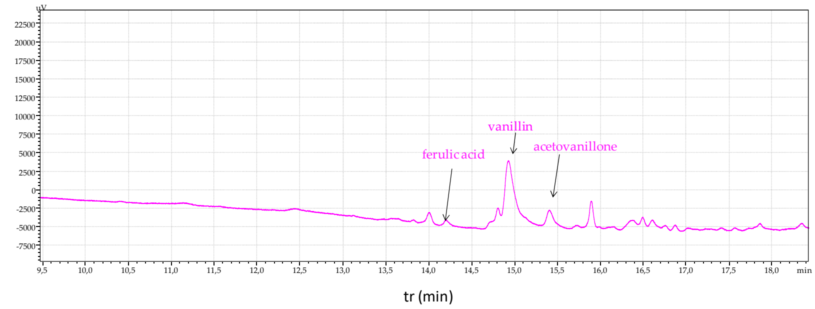

Figure S7. HPLC chromatograms: for microwave depolymerized river cane lignin.

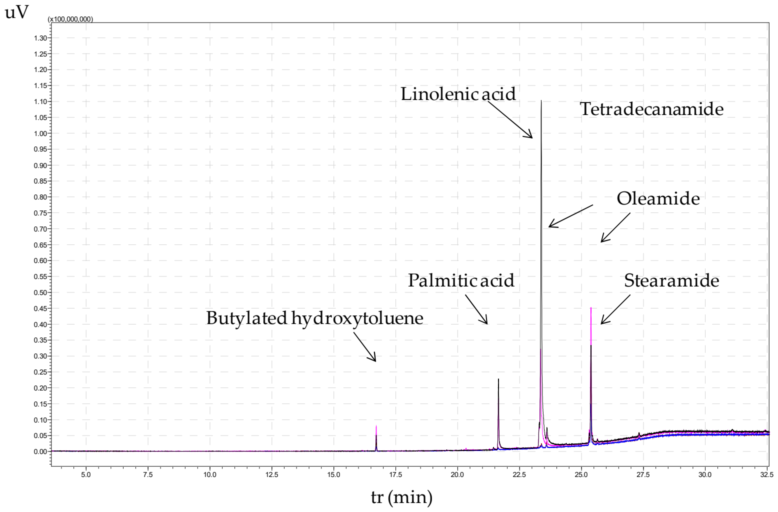

Figure S8. GC/MS chromatogram for lignin depolymerization of other waste lignin with microwave, (ethyl acetate extract). Rice husk (blue line), broccoli (pink line), wheat straw (brown line) and olive stones (black line).
